# Supplementary material for: Biology and Bias in Cell Type-Specific RNAseq of Nucleus Accumbens Medium Spiny Neurons
Source: Sci Rep. 2019 Jun 6;9:8350. doi: 10.1038/s41598-019-44798-9 (PMC6554355; doi:10.1038/s41598-019-44798-9)
Supplement: Supplementary file 1 — Supplementary Information [file 41598_2019_44798_MOESM1_ESM.pdf]

Biology and Bias in Cell Type-Specific RNAseq of Nucleus Accumbens Medium Spiny Neurons

Kronman, Hope[1]* & Richter, Felix[2,3]*, Labonté, Benoit[1], Chandra, Ramesh[4], Zhao, Shan[5], Hoffman, Gabriel[3], Lobo, Mary Kay[4], Schadt, Eric E.[3], Nestler, Eric J.[1#]

* These authors contributed equally to this work
[#] Corresponding author, e-mail: eric.nestler@mssm.edu
[1] Dept of Neuroscience and Friedman Brain Institute, Icahn School of Medicine at Mount Sinai
[2] Dept of Pediatrics, Icahn School of Medicine at Mount Sinai
[3] Dept of Genetics and Genomic Sciences, Icahn School of Medicine at Mount Sinai
[4] Dept of Anatomy and Neurobiology, University of Maryland School of Medicine
[5] Dept of Anesthesiology, Icahn School of Medicine at Mount Sinai

Supplementary figure legends

Supplementary Figure 1. Differences in genomic distribution and differential expression across methods.

(A) Percent of intronic reads across samples for each gene, median indicated as a line in method color
(B) Differential expression overlap using lenient filters on all (1 FPM in at least two samples)
(C) Differential expression overlap using strict filters on all (FPKM $\geq$ 1 in at least one sample and all samples with FPKM < 5.0×10^4)
(D) Differential expression overlap using strict filter for whole cell and nuclear and a less strict filter for RiboTag (FPKM $\geq$ 0.1 in at least two samples and all samples with FPKM < 5.0×10^4)
(E) Density plots of gene lengths for all genes, protein-coding genes, and non-coding genes for each method (whole cell = black, nuclear = blue, RiboTag = red)

Supplementary Figure 2. Hub network overlaps and nuclear DET-predicted TFBSs

(A) Overlap of hub network genes for D1- and D2-correlated modules across method
(B) Overlap of genes showing regulation by transcription, cytosolic mechanisms, or translation in D1- and D2-MSNs. 0 overlaps are not labeled.
(C) List of 27 miRNAs predicted upstream of the (Transcription D1) / (Cytosol D2) overlap (overlap p-value < 0.05) and FPKM values of the 4 miRNAs from this list that are differentially expressed in D2-nuclei (but not in D2-whole cells)

Supplementary Figure 3. Variance in just genes detected in all three methods.

(A) PCA of combined data showing separation of samples by cell type on PC5/PC6. Key is shown in figure
(B) PCA of nuclear, whole cell, and RiboTag datasets from this study along with RiboTag RNA-sequencing from Song et al, 2018. Key is shown in figure
(C) Variance explained by method, cell type and residuals across the transcriptome. Central bars represent median

Supplementary Figure 4. Hub network overlaps and nuclear DET-predicted TFBSs

(D) Overlap of hub network genes for D1- and D2-correlated modules across method
(E) Overlap of predicted TFBSs for D2 whole cell and RiboTag hub networks with predicted TFBSs for nuclear D2-DEGs
(F) Overlap of predicted TFBSs for D1 whole cell and RiboTag hub networks with predicted TFBSs for nuclear D1-DEGs

Supplementary Figure 5. Variance distributions for D1- and D2-DETs across methods.

(A) Variance distribution for nuclear D1- and D2-DEGs (top of the box = 3rd quartile, midline = median, bottom of the box = 1st quartile)

(B) Variance distribution for RiboTag D1- and D2-DEGs (top of the box = 3rd quartile, midline = median, bottom of the box = 1st quartile)
(C) Variance distribution for whole cell D1- and D2-DEGs (top of the box = 3rd quartile, midline = median, bottom of the box = 1st quartile)

Supplementary Figure 6. FACS plots demonstrating gating strategy.

(A) Gating strategy for D1-whole cells. From the left, selecting whole neurons (SSC vs. FSC), selecting live neurons (DAPI vs. FSC), selecting GFP-negative, tdTomato-positive whole neurons (GFP vs. Texas Red)
(B) Gating strategy for D2-whole cells. From the left, selecting whole neurons (SSC vs. FSC), selecting live neurons (DAPI vs. FSC), selecting GFP-positive, tdTomato-negative whole neurons (GFP vs. Texas Red)
(C) Gating strategy for D1-nuclei. From the left, selecting nuclei (SSC vs. FSC), selecting GFP-positive nuclei (GFP vs. FSC)
(D) Gating strategy for D2-nuclei. From the left, selecting nuclei (SSC vs. FSC), selecting GFP-positive nuclei (GFP vs. FSC)

Supplementary table list

Supplementary Table 1: Nuclear DEG logistic regression
Supplementary Table 2: Differential expression by method
Supplementary Table 3: FPKM by method
Supplementary Table 4: FPKM and variance correlations across methods
Supplementary Table 5: Variance explained by gene
Supplementary Table 6: Biotypes of genes from variance partitioning analysis
Supplementary Table 7: gProfiler enrichments
Supplementary Table 8: Genes enriched in each method
Supplementary Table 9: GWAS risk loci overlaps

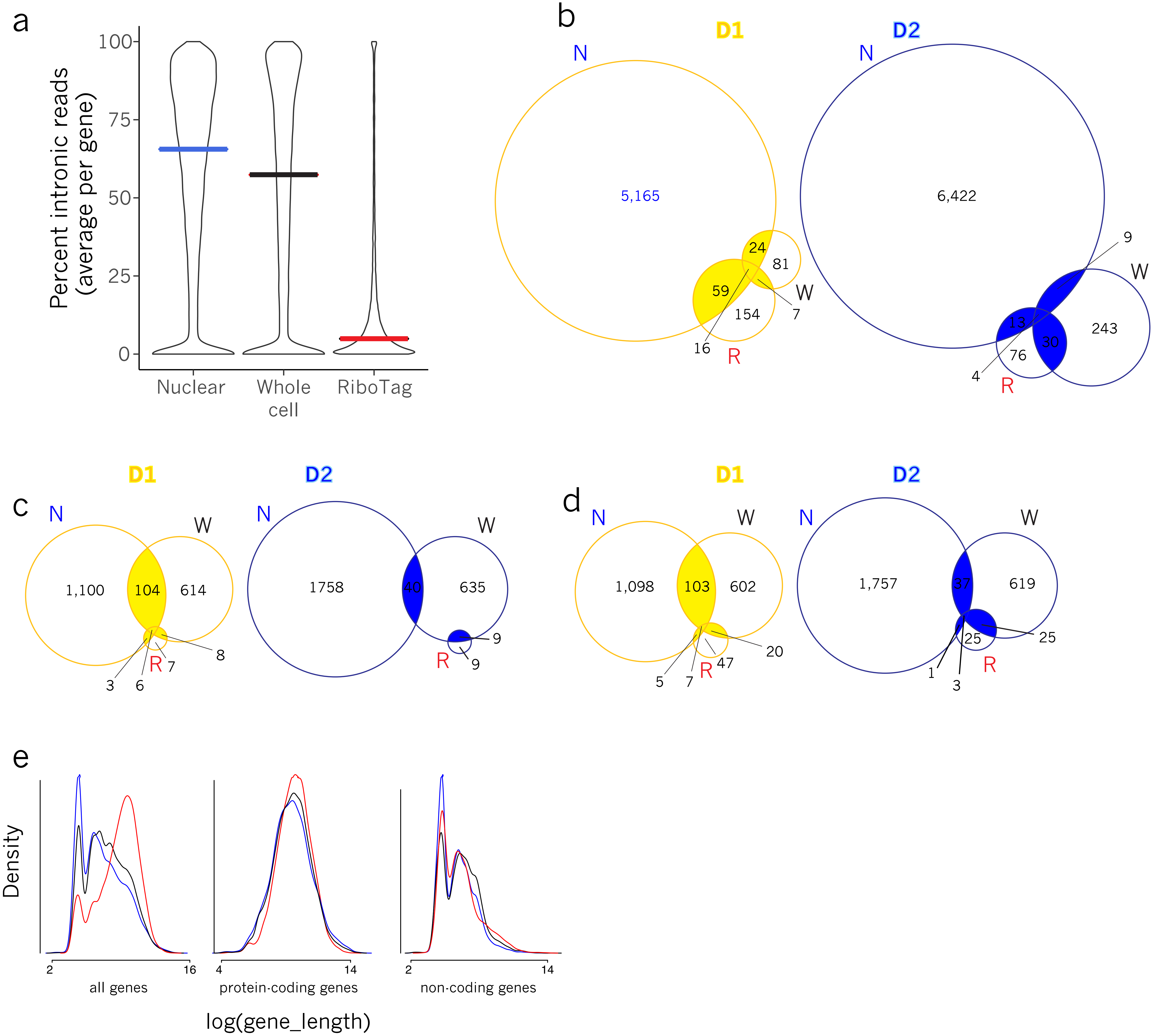
a
Percent intronic reads (average per gene)
100
75
50
25
0
Nuclear
Whole cell
RiboTag
b
D1
D2
N
W
R
5,165
24
81
59
154
7
16
6,422
9
13
30
243
76
4
c
1,100
104
614
8
7
3
6
1758
40
635
9
9
d
1,098
103
602
20
47
5
7
1,757
37
619
25
25
1
3
e
Density
2
16
4
14
2
14
all genes
protein-coding genes
non-coding genes
log(gene_length)

a

$$y_{expr} = \beta_0 + \beta_1 x_{d2} + \beta_2 x_{ribo} + \beta_3 x_{wc} + \beta_4 x_{wc} \cdot x_{d2} + \beta_5 x_{ribo} \cdot x_{d2}$$

Sequencing method…	Measures changes in…	Relevant independent variables are… x_{d2}	x_{ribo}	x_{wc}	Coefficients being solved for are…
Nuclear	**Transcription**	0 and 1	0	0	β_1
WC	**Transcription** + **Post-transcriptional processes**	0 and 1	0	1	$\beta_1 + \beta_4$
RiboSeq	**Transcription** + **Post-transcriptional processes**+ **Translation**	0 and 1	1	0	$\beta_1 + \beta_5$

Therefore, genes with significant…	Have differential…
$\boldsymbol{\beta_1}$	**Transcription**
$(\beta_1 + \beta_4) - \beta_1 = \boldsymbol{\beta_4}$	**Post-transcriptional processes**
$(\beta_1 + \beta_5) - (\beta_1 + \beta_4) = \boldsymbol{\beta_5 - \beta_4}$	**Translation**

b

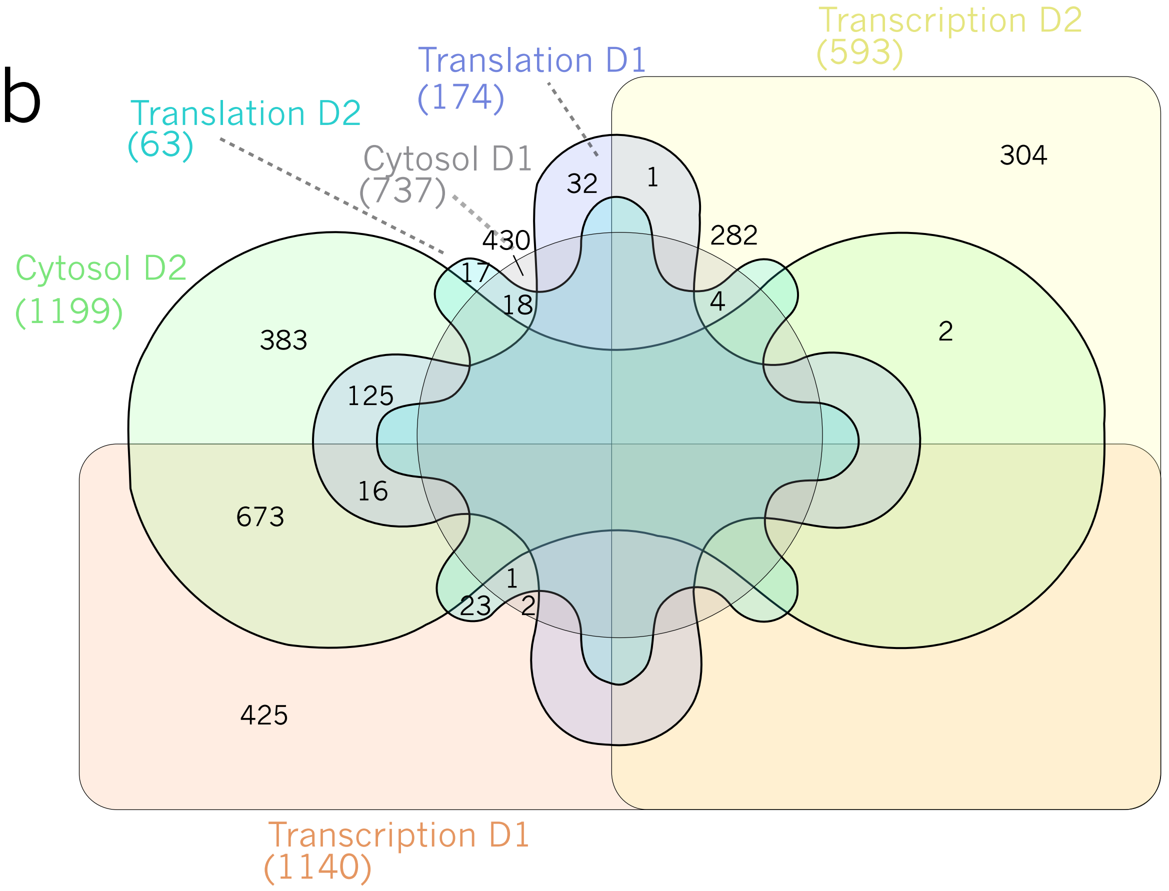

c

mmu-miR-9-5p	mmu-miR-301b-3p
mmu-miR-7b-5p	mmu-miR-297a-5p
mmu-miR-7a-2-3p	mmu-miR-26a-5p
mmu-miR-7a-1-3p	mmu-miR-24-3p
mmu-miR-758-3p	mmu-miR-223-3p
mmu-miR-669d-5p	mmu-miR-211-5p
mmu-miR-541-5p	mmu-miR-19b-3p
mmu-miR-539-5p	mmu-miR-199a-3p
mmu-miR-490-3p	mmu-miR-188-5p
mmu-miR-425-5p	mmu-miR-181a-5p
mmu-miR-384-3p	mmu-miR-15a-5p
mmu-miR-340-5p	mmu-miR-149-5p
mmu-miR-30e-5p	mmu-let-7b-5p

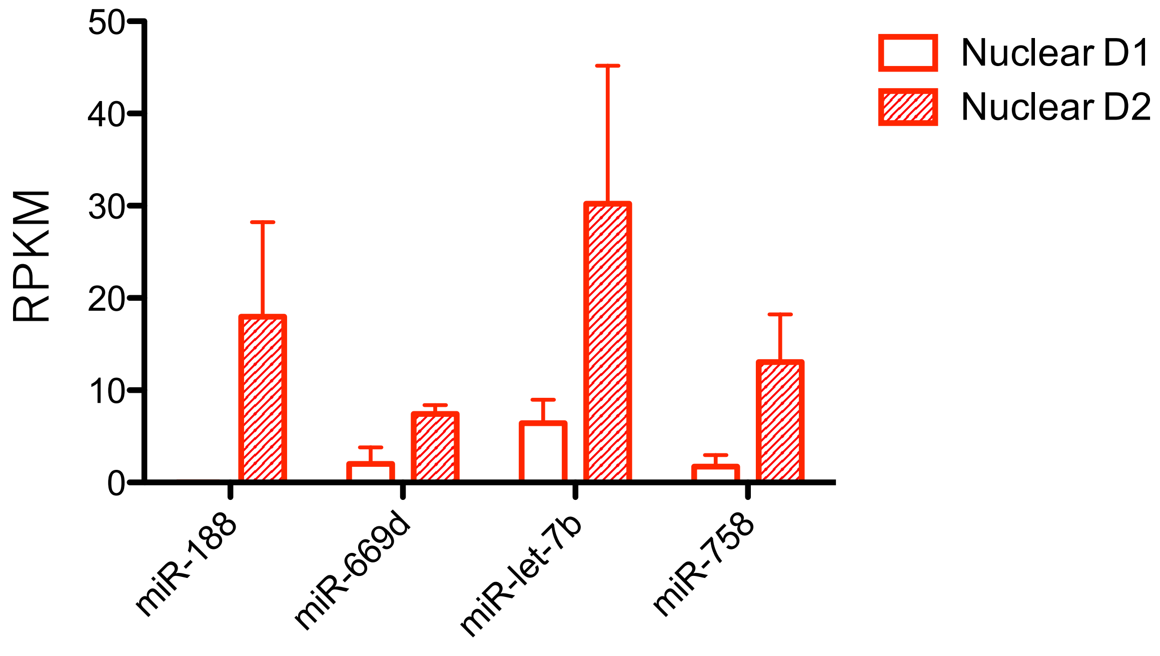

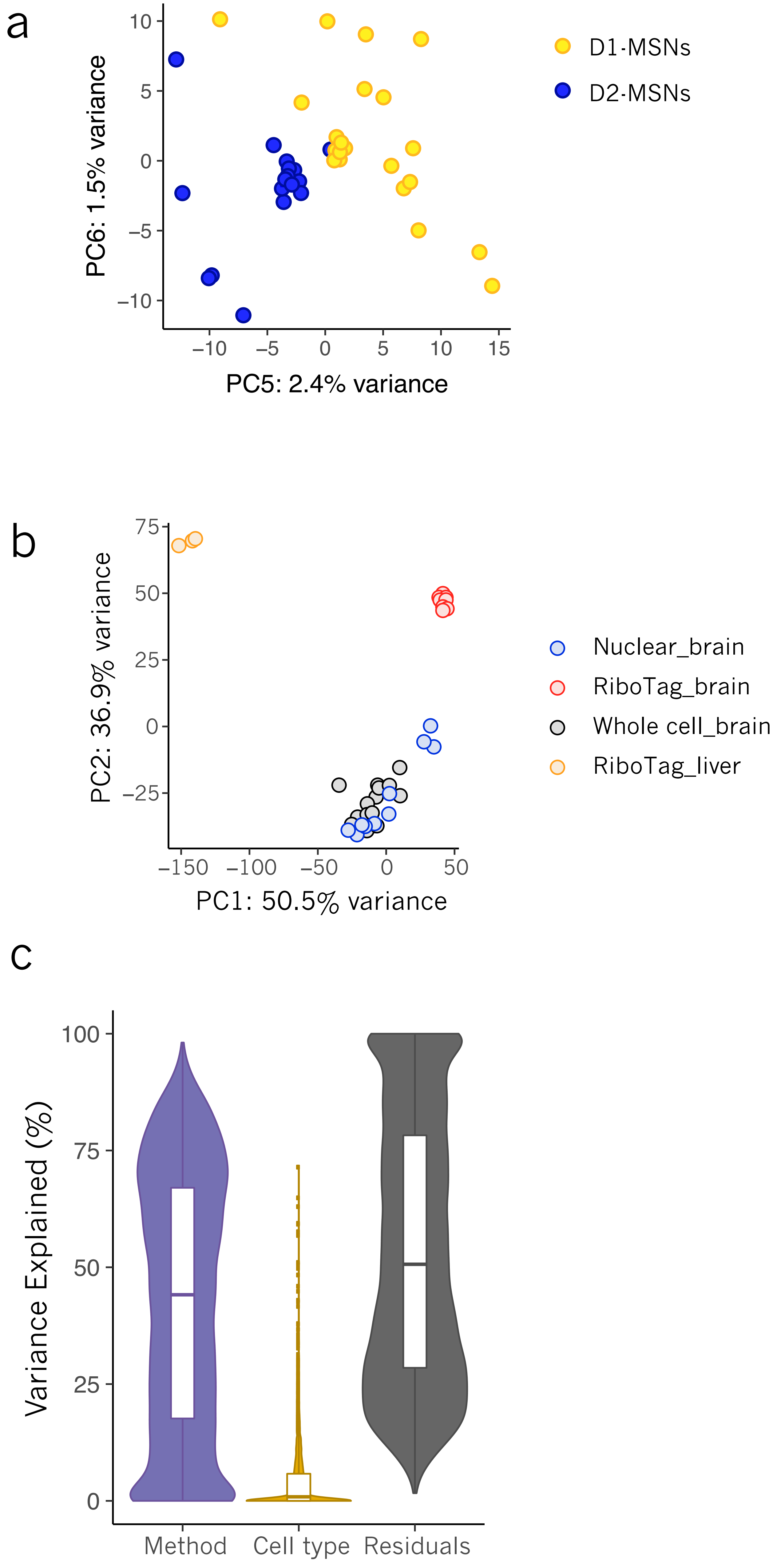
a
PC6: 1.5% variance
PC5: 2.4% variance
D1-MSNs
D2-MSNs
b
PC2: 36.9% variance
PC1: 50.5% variance
Nuclear_brain
RiboTag_brain
Whole cell_brain
RiboTag_liver
c
Variance Explained (%)
Method
Cell type
Residuals

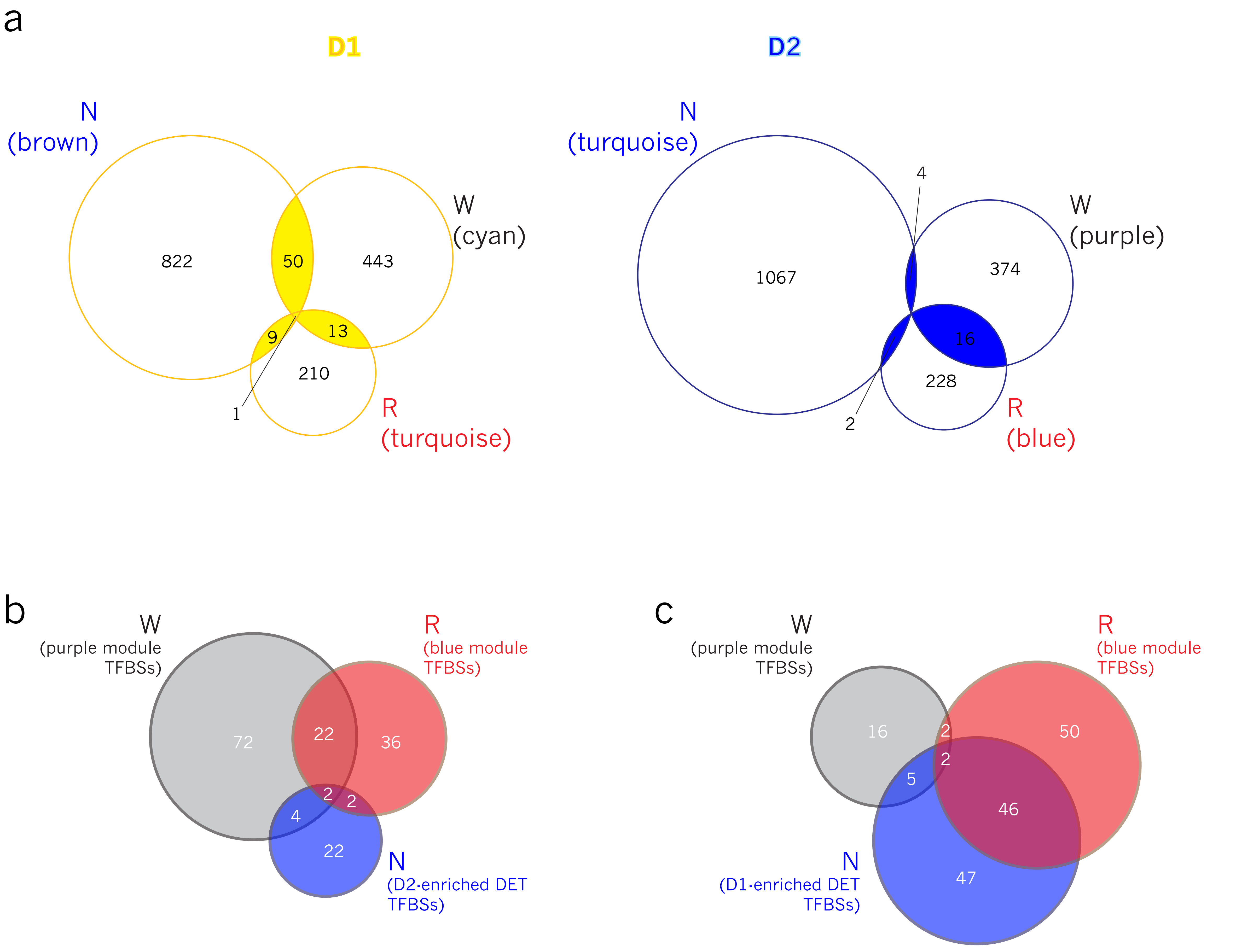
a
D1
N
(brown)
W
(cyan)
R
(turquoise)
822
50
443
9
13
210
1
D2
N
(turquoise)
W
(purple)
R
(blue)
1067
4
374
16
228
2
b
W
(purple module
TFBSs)
R
(blue module
TFBSs)
N
(D2-enriched DET
TFBSs)
72
22
36
2
2
4
22
c
W
(purple module
TFBSs)
R
(blue module
TFBSs)
N
(D1-enriched DET
TFBSs)
16
2
50
2
5
46
47

a

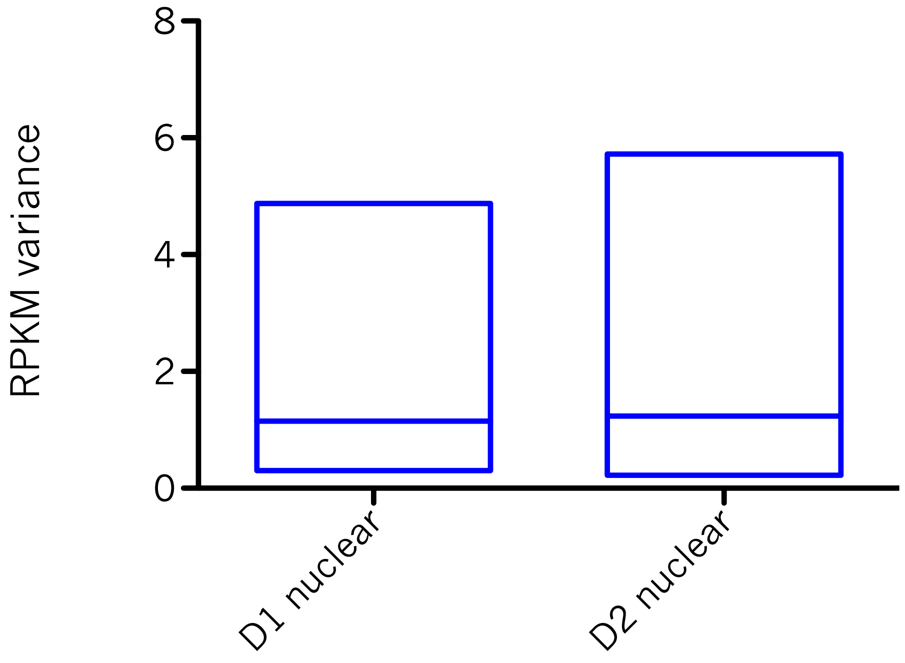

RPKM variance
8
6
4
2
0
D1 nuclear
D2 nuclear

b

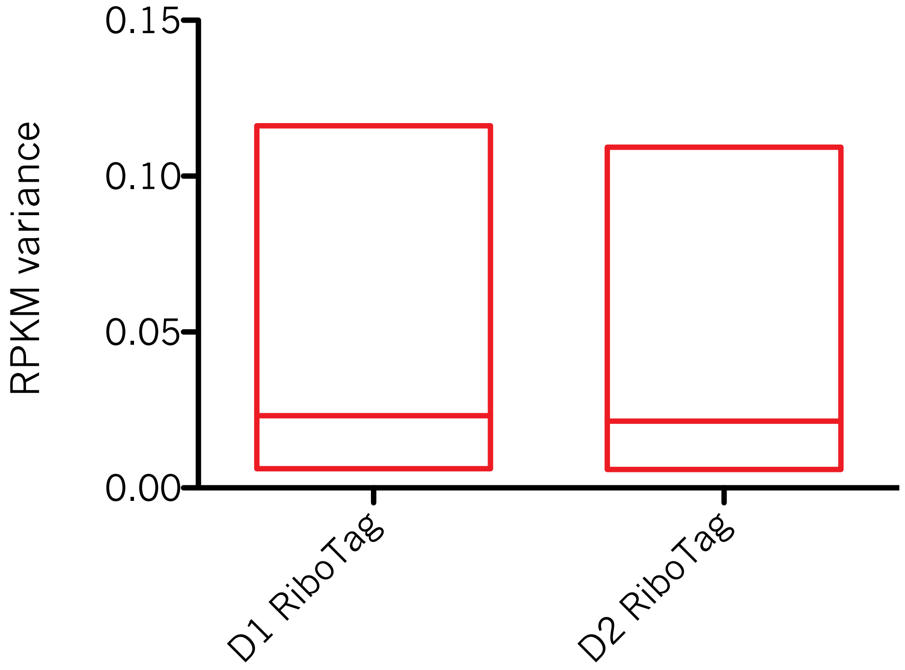

RPKM variance
0.15
0.10
0.05
0.00
D1 RiboTag
D2 RiboTag

c

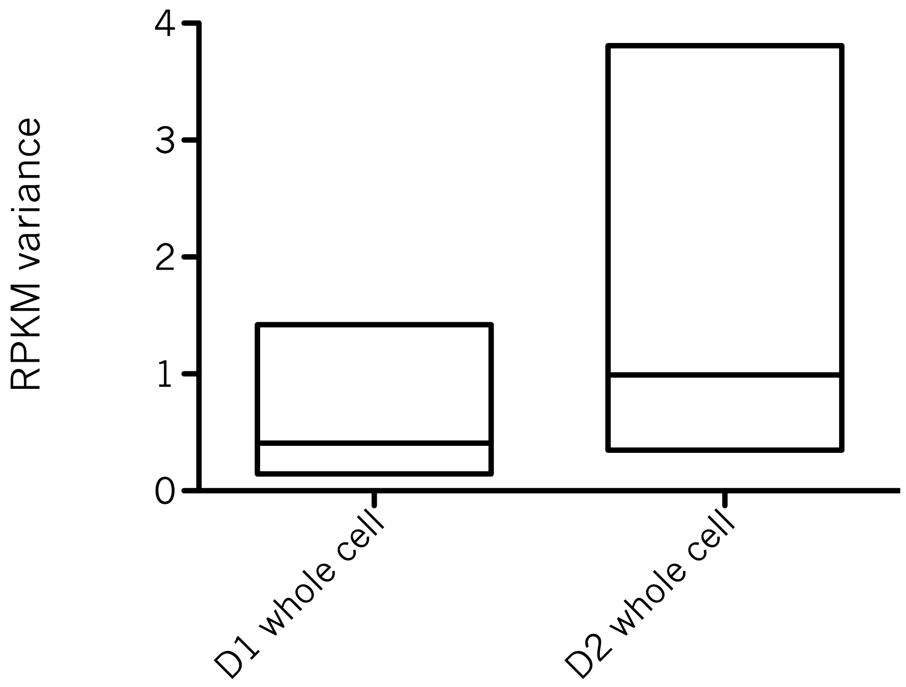

RPKM variance
4
3
2
1
0
D1 whole cell
D2 whole cell

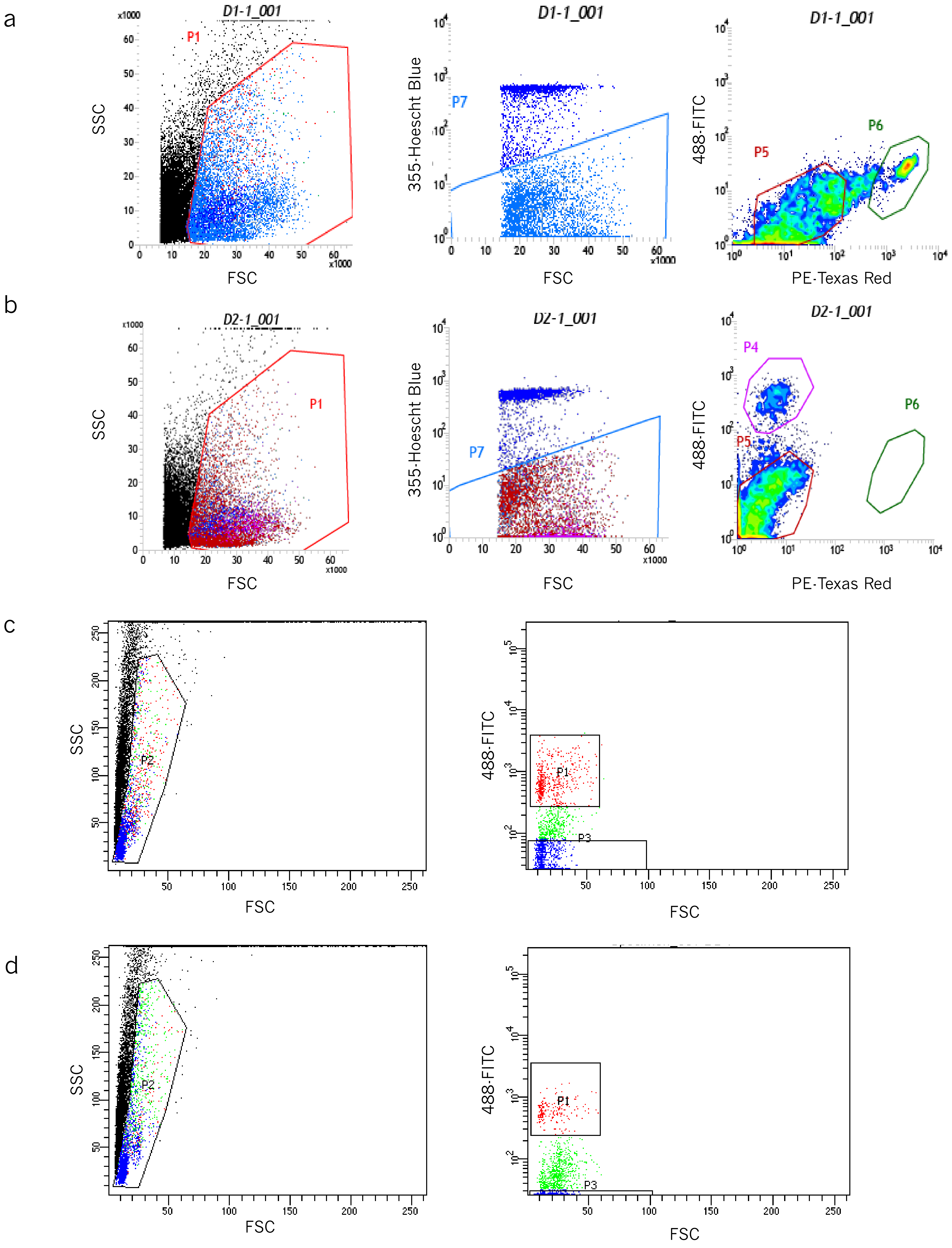

a
D1-1_001
P1
SSC
FSC
355-Hoescht Blue
P7
488-FITC
P5
P6
PE-Texas Red
b
D2-1_001
P4
c
P2
P3
d
